# Managing Cataract in a Myopic Eye with Iridocorneal Endothelial Syndrome and Horizontal Nystagmus: a Rare Clinical Association

**DOI:** 10.22336/rjo.2026.25

**Published:** 2026

**Authors:** Paul-Gabriel Borodi, Iulia-Maria Gavriș, Al Masri Malaz, Maria-Monica Gavriș

**Affiliations:** 1 IOSUD Doctoral School, “George Emil Palade” University of Medicine, Pharmacy, Science, and Technology of Târgu Mureș, Mureş, Romania; 2 Faculty of Medicine, Victor Babeș University of Medicine and Pharmacy, Timișoara, Romania; 3 Ophthalmology Department, “Dr. Constantin Papilian” Military Emergency Hospital Cluj-Napoca, Romania

**Keywords:** iridocorneal endothelial syndrome, myopia, nystagmus, cataract surgery, ICE = Iridocorneal endothelial syndrome, IOL = Intraocular lens

## Abstract

**Objectives:**

To report a rare clinical association of iridocorneal endothelial (ICE) syndrome with high myopia, horizontal nystagmus, and cataract, and to highlight the preoperative and intraoperative considerations required to achieve successful cataract surgery in such complex cases.

**Case report:**

A 41-year-old female presented with progressive visual deterioration. Examination revealed horizontal nystagmus, narrow palpebral fissures, corneal opacities, iris atrophic changes with superotemporal corectopia, and significant cortico-nuclear lens opacities. Best-corrected visual acuity was 1/50 in the right eye and 2/50 in the left eye, with intraocular pressures within normal limits. The patient underwent phacoemulsification with a foldable hydrophobic acrylic IOL implanted in the capsular bag. Retrobulbar anesthesia stabilized the globe; four iris retractors were used for inadequate pupillary dilation, and trypan blue facilitated capsulorhexis. The soft-shell technique and reduced phacoemulsification parameters were employed to protect the corneal endothelium. No intraoperative complications occurred. At one month postoperatively, BCVA was 2/50 with stable refraction (-2.75 D) and no corneal decompensation.

**Discussion:**

ICE syndrome complicates cataract surgery due to endothelial compromise, iris abnormalities, and the risk of intraoperative complications. Individualized surgical strategies, including globe stabilization, pupil expansion, careful endothelial protection, and modified phacoemulsification parameters, were critical to achieving a favorable outcome.

**Conclusion:**

Cataract surgery in patients with ICE syndrome complicated by high myopia and horizontal nystagmus is feasible with meticulous preoperative assessment and tailored intraoperative techniques. Favorable visual outcomes can be achieved, but long-term follow-up is essential to monitor for progressive glaucoma and corneal decompensation.

## Introduction

Iridocorneal endothelial (ICE) syndrome is an uncommon condition characterized by abnormal proliferation and structural changes of the corneal endothelium, leading to a gradual obstruction of the iridocorneal angle and iris alterations, including atrophy and the formation of iris defects. These pathological changes frequently result in corneal decompensation and secondary glaucoma, which are the leading causes of visual impairment in affected individuals. ICE syndrome encompasses a range of clinical variants, namely progressive essential iris atrophy, Cogan–Reese syndrome, and Chandler syndrome [1,2].

In Chandler syndrome, the iris shows areas of atrophy without full-thickness holes, and the pupil may be displaced from its normal central position, a condition known as corectopia. The cornea is affected early, with marked edema and endothelial dystrophy, and ICE cells can be observed on confocal microscopy. The anterior chamber angle develops peripheral anterior synechiae [3].

In the progressive iris atrophy subtype, the iris shows full-thickness holes, and multiple pupils (polycoria) often develop. Corneal involvement may include endothelial dystrophy and edema, with ICE cells visible on confocal microscopy, and the anterior chamber angle also shows peripheral anterior synechiae [4].

Cogan-Reese syndrome is characterized by nodules on the iris and areas of atrophy, though pupil changes are uncommon. The cornea may develop endothelial dystrophy and edema, with ICE cells detectable on confocal microscopy, and the anterior chamber angle demonstrates peripheral anterior synechiae as well [5].

ICE syndrome typically presents as a unilateral condition, most commonly affecting women aged between 30 and 50 years. Molecular studies using polymerase chain reaction (PCR) have often detected herpes simplex virus DNA, supporting the hypothesis of a possible viral etiology [6].

The study aims to describe the difficulties that may arise after phacoemulsification and intraocular lens (IOL) implantation in patients with ICE syndrome who also have myopia and horizontal nystagmus.

## Case report

**Fig. 1:**
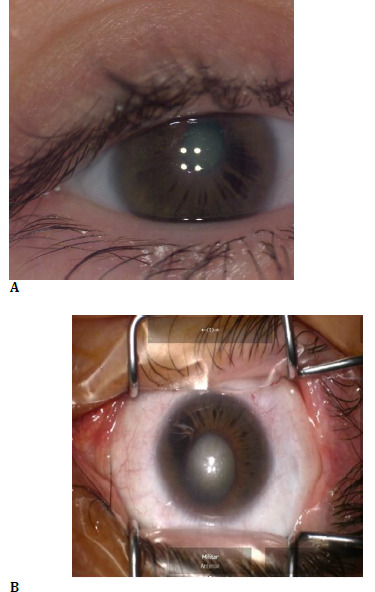
**A, B** Anterior segment of the right eye

**Fig. 2:**
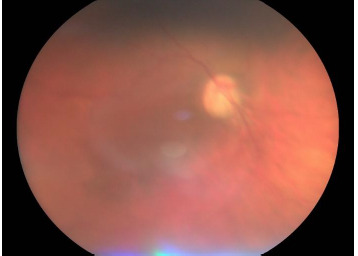
Posterior segment of the right eye

A 41-year-old female presented with progressive visual deterioration. Her family history was notable for high myopia and astigmatism in her father and glaucoma in her mother. On examination, the right eye exhibited horizontal nystagmus and narrow palpebral fissures. The cornea showed a deep, round central opacity with peripheral limbal vascularization. The iris displayed radial atrophic areas with superotemporal corectopia. Lens examination revealed grade III cortico-nuclear opacities. Fundus evaluation demonstrated a tilted optic disc with pallor, reduced-caliber retinal vessels, and a thinned, attenuated retina (**Fig. 1 A, B, Fig. 2**).

The left eye also presented with horizontal nystagmus and narrow palpebral fissures. The cornea was clear, while the iris showed atrophic areas. Cortico-nuclear opacities were graded II/III. Fundus examination revealed a tilted optic disc, reduced-caliber retinal vessels, and a thinned retina.

Best-corrected visual acuity was 1/50 in the right eye and 2/50 in the left eye. Intraocular pressure measured 18 mmHg in the right eye and 20 mmHg in the left eye. Gonioscopy of the right eye revealed superior-nasal peripheral anterior synechiae. Refraction of the left eye was -5.75/-13.00/1. Ultrasound contact biometry showed axial lengths of 25.47 mm in the right eye and 25.69 mm in the left eye.

The patient’s presentation was consistent with ICE syndrome - Chandler subtype associated with myopia and horizontal nystagmus, complicated by significant lens opacities, corneal changes, and structural retinal alterations.

Phacoemulsification surgery was planned for the right eye. Given the patient’s myopia, a postoperative target of -2.50 D myopia was set. IOL power was selected based on the Holladay formula. To control the nystagmus during surgery, a retrobulbar anesthesia was performed. To protect the corneal endothelium, the soft-shell technique was utilized. Due to inadequate pupillary dilation, four iris retractors were placed before performing the capsulorhexis. The anterior capsule was stained with trypan blue due to the whitish tint of the opacities and to facilitate handling of the elastic capsule in this young patient. The lens nucleus was divided using the stop-and-chop method. Reduced phacoemulsification parameters were used to minimize anterior chamber fluctuations in this myopic patient. A foldable hydrophobic acrylic monobloc IOL was then implanted into the capsular bag. The main scleral incision was closed with a 10/0 Nylon suture, and antibiotics were instilled subconjunctivally before concluding the surgery. No intraoperative complications occurred.

**Fig. 3:**
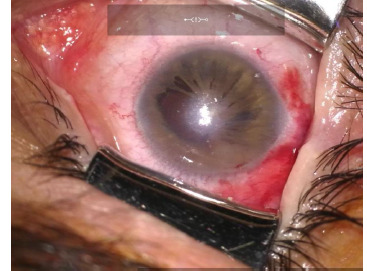
Anterior segment of the right eye postoperatively

On the first postoperative day, BCVA in the right eye was 2/50. At one month postoperatively, BCVA remained 2/50, and IOP was 21 mmHg. No corneal decompensation was observed, and the final refraction stabilized at -2.75 D (**Fig. 3**).

## Discussion

Cases of ICE syndrome associated with cataracts, myopia, and nystagmus are rare in our daily practice. We presented this case to highlight the special considerations required to successfully perform cataract surgery in these patients.

In the literature, cataract surgery in patients with ICE syndrome is described as particularly challenging due to corneal endothelial compromise, iris abnormalities, and a higher risk of intraoperative complications. Al-Humimat et al. highlighted these difficulties in their case report, emphasizing meticulous intraoperative management, careful endothelial protection, and the use of pupil expansion techniques to manage small or irregular pupils. While both our approach and that of Al-Humimat et al. prioritized endothelial preservation and controlled surgical maneuvers, our case also required management of nystagmus and significant lens opacities, underscoring the need for individualized strategies tailored to the anatomical and functional challenges of ICE syndrome [7].

Preoperative measurements were challenging due to the patient’s horizontal nystagmus, which limited the precision of optical biometry. Contact ultrasound biometry was used to obtain reliable axial length measurements and achieve the desired refractive outcome. Although specular microscopy was not performed, it would have been useful for monitoring the corneal endothelium in this patient with ICE syndrome.

## Conclusion

In conclusion, this case highlights that successful cataract surgery in patients with ICE syndrome complicated by high myopia and horizontal nystagmus is feasible with careful preoperative planning and individualized intraoperative strategies. Thorough assessment, meticulous endothelial protection, and globe stabilization are key to achieving favorable visual outcomes. Despite good short-term results, long-term monitoring is essential to detect and manage potential complications such as glaucoma and corneal decompensation.
